# Understanding the Cures Act Information Blocking Rule in cancer care: a mixed methods exploration of patient and clinician perspectives and recommendations for policy makers

**DOI:** 10.1186/s12913-023-09230-z

**Published:** 2023-03-06

**Authors:** Joanna Veazey Brooks, Carli Zegers, Christian T. Sinclair, Elizabeth Wulff-Burchfield, Amanda R. Thimmesch, Daniel English, Heather V. Nelson-Brantley

**Affiliations:** 1 University of Kansas School of Medicine, 3901 Rainbow Boulevard, Kansas City, KS 66160 USA; 2 University of Kansas School of Nursing, 3901 Rainbow Boulevard, Kansas City, KS 66160 USA

**Keywords:** 21^st^ Century Cures Act, Cancer care, Information Blocking Rule, Open Notes, Patient-clinician communication, Policy implementation

## Abstract

**Background:**

The 21^st^ Century Cures Act Interoperability and Information Blocking Rule was created to increase patient access to health information. This federally mandated policy has been met with praise and concern. However, little is known about patient and clinician opinions of this policy within cancer care.

**Methods:**

We conducted a convergent parallel mixed methods study to understand patient and clinician reactions to the Information Blocking Rule in cancer care and what they would like policy makers to consider. Twenty-nine patients and 29 clinicians completed interviews and surveys. Inductive thematic analysis was used to analyze the interviews. Interview and survey data were analyzed separately, then linked to generate a full interpretation of the results.

**Results:**

Overall, patients felt more positive about the policy than clinicians. Patients wanted policy makers to understand that patients are unique, and they want to individualize their preferences for receiving health information with their clinicians. Clinicians highlighted the uniqueness of cancer care, due to the highly sensitive information that is shared. Both patients and clinicians were concerned about the impact on clinician workload and stress. Both expressed an urgent need for tailoring implementation of the policy to avoid unintended harm and distress for patients.

**Conclusions:**

Our findings provide suggestions for optimizing the implementation of this policy in cancer care. Dissemination strategies to better inform the public about the policy and improve clinician understanding and support are recommended. Patients who have serious illness or diagnoses such as cancer and their clinicians should be included when developing and enacting policies that could have a significant impact on their well-being. Patients with cancer and their cancer care teams want the ability to tailor information release based on individual preferences and goals. Understanding how to tailor implementation of the Information Blocking Rule is essential for retaining its benefits and minimizing unintended harm for patients with cancer.

**Supplementary Information:**

The online version contains supplementary material available at 10.1186/s12913-023-09230-z.

## Background

On April 5^th^, 2021 the 21^st^ Century Cures Act Interoperability and Information Blocking Rule (Information Blocking Rule) went into effect, requiring health care clinicians to give patients rapid access to all clinical notes and medical test results [1]. Research has shown that having access to clinical notes and test results can increase patient participation in their care [2, 3], improve patients’ understanding of their health conditions [2–5], and provide insight into healthcare decision making [3, 6].

However, early research also shows that these benefits do not extend universally to all patients and all health conditions. Some patients with cancer and other emotionally charged conditions have reported increased anxiety and confusion after reading test results and the notes of their care clinicians [4]. Clinicians also have expressed concern [3] that patient access to clinical notes and test results increases clinician workload [5–8] requiring that they respond to patient questions at all hours and requests for edits to their health record. Additionally, clinicians report concern that patients may lack adequate knowledge of medical terminology to effectively understand clinical notes and test results [7, 8].

In cancer care, the immediate release of information conveying a cancer diagnosis or prognosis has the potential to cause undue stress and anxiety for a patient before a care clinician is able to explain medical terms and devise a plan of treatment. However, there is currently a gap in knowledge on the experiences of patients and clinicians of the Information Blocking Rule in cancer care. This article describes patient and clinician responses to the Information Blocking Rule and their messages to policy makers.

## Methods

### Study design and research methods

We used a convergent parallel mixed methods design [9] to understand patient and clinician reactions to the Information Blocking Rule. A convergent parallel mixed methods design was selected to achieve a more complete understanding of the phenomenon by synthesizing complementary qualitative and quantitative findings. In line with the convergent parallel design, quantitative data and qualitative data were collected during the same phase of the research process, were prioritized equally, analyzed independently, then mixed during the overall interpretation phase. [9] This study, which reports on respondents’ messages for policy makers, was part of a larger study that investigated patient and clinician experiences of the Information Blocking Rule. See Supplemental File for full study surveys and interview guides. Here, we specifically report on two research questions: (1) What are patients with cancer and cancer care clinician opinions of the Information Blocking Rule; and (2) What would patients with cancer and cancer care clinicians say to policy makers—what would they like policy makers to know?

### Data collection

Patient and clinician participants were recruited from ambulatory and inpatient oncology and oncology palliative care settings within one Midwestern National Cancer Institute-designated comprehensive cancer care center. Purposive maximum variation sampling [10] was used to recruit a variety of clinicians (physicians, advanced practice providers, and registered nurses) and patients with a variety of cancer types (e.g., breast, colon, heme, gynecological, prostate, lung, brain). Patient and clinician participants completed an electronic survey via REDCap [11, 12] and an individual, semi-structured interview ranging from 28–75 min. Questions specific to this study are presented in Table 1. Participants received $50 for participating in the study. Recruitment continued until data saturation was reached. Interviews were recorded and transcribed verbatim by a professional transcription service.

**Table 1:** Survey and Interview Guide Questions

	**Survey Question**	**Response Options**
**Patient Survey**	What is your overall opinion of the 21^st^ Century Cures Act Interoperability and Information Blocking Rule that requires health care providers (such as doctors and nurses) to provide patients with on-demand access to their clinical notes?	- Strongly support - Somewhat support - Neutral - Somewhat oppose - Strongly oppose - Not sure - Never heard of it
**Clinician Survey**	What is your overall opinion of the 21^st^ Century Cures Act Interoperability and Information Blocking Rule that requires clinicians to provide patients with on-demand access to your clinical notes?	- Strongly support - Somewhat support - Neutral - Somewhat oppose - Strongly oppose - Not sure - Never heard of it
	**Interview Guide Question**	**Follow up probes**
**Patient Interview Guide**	Are you familiar with the 21^st^ Century Cures Act Interoperability and Information Blocking Rule? [if no, provide brief overview] How do you feel about the policy?	What, if anything, would you want your legislator to know? If you could share anything about this new policy, what would it be?
**Clinician Interview Guide**	Are you familiar with the 21^st^ Century Cures Act Interoperability and Information Blocking Rule? [if no, provide brief overview] How do you feel about the policy?	What do you want policy makers to know? If you could say anything to them about this new regulation, what would it be? In what ways, if at all, has your process of informing patients of diagnosis or discussing test results, treatment options and regimens changed now that the policy is in place?

### Data analysis

Interview and survey data were first analyzed separately, then linked to generate a full interpretation of the findings [9]. The mixing of data was of the linking type [13], whereby the quantitative survey data and qualitative interview data were juxtaposed to each other but not merged, as a mechanism for extending the knowledge ascertained from each. We used an inductive thematic analysis approach to analyze the qualitative interview data [14]. Multiple members of our study team participated in coding, and identification of themes, with varied perspectives and closeness to the data, which bolsters the credibility and confirmability of our findings [15]. Descriptive statistics were used to analyze survey data and the analysis was completed on items specific to the purpose of this study.

## Results

Twenty-nine patients completed the survey and interview and 29 clinicians completed the survey and interview. Table 2 outlines participant characteristics.

**Table 2:** Participant characteristics

**Demographic**	**Patient (*N* = 29)** Number(%)	**Clinician (*N* = 29)** Number(%)
Age range		
20–35 years	3(0.3%)	5(17.2%)
36–50 years	7(24.1%)	17(58.6%)
51–65 years	14(48.3%)	7(24.1%)
65 + years	5(17.2%)	0(0.0%)
Gender		
Female	25(86.2%)	25(86.2%)
Male	4(13.8%)	4(13.8%)
Transgender	0(0.0%)	0(0.0%)
Non-binary	0(0.0%)	0(0.0%)
Ethnicity		
Non-Hispanic	19(65.5%)	27(93.1)
Hispanic: Mexican	2(6.9%)	0(0.0%)
Hispanic: Puerto Rican	0(0.0%)	0(0.0%)
Hispanic: El Salvadorian	0(0.0%)	0(0.0%)
Hispanic: Other	5(17.2%)	0(0.0%)
Prefer not to say	0(0.0%)	1(3.4%)
Race		
White/Caucasian	19(65.5%)	27(93.1%)
Black/African American Asian	6(20.7%)	0(0.0%)
Native Hawaiian or Pacific Islander	2(6.9%)	0(0.0%)
Native American or Alaskan Native	0(0.0%)	0(0.0%)
Other	0(0.0%)	0(0.0%)
Prefer not to say	1(3.4%)	1(3.4%)
Highest level of education		
High school diploma or GED	2(6.9%)	
Vocational/Technical Training	2(6.9%)	
Associate degree	4(13.8%)	
Bachelor’s degree	13(44.8%)	
Master’s degree	5(17.2%)	
Doctorate	2(6.9%)	
I did not complete high school or GED	1(3.4%)	
Prefer not to say	0(0.0%)	
Cancer Care Received (Select all that apply)		
Chemotherapy	16(55%)	
Radiation	14(48%)	
Surgery	15(52%)	
Palliative care	7(24%)	
Monitoring on a schedule	17(59%)	
Immunotherapy	7(24%)	
Stem cell/bone marrow transplant	1(3%)	
BiTE/BiKE/TriKE treatments	0(0%)	
Tumor-infiltrating lymphocyte (TIL) treatment	0(0%)	
Other	6(21%)	
Professional type		
Physician (MD, DO)		9(31.0%)
Advanced Practice Provider (APRN, PA)		6(20.6%)
Registered Nurse		14(48.3%)
Years in practice		
< 1 year		0(0.0%)
1–5 years		2(6.9%)
5–10 years		10(34.5%)
10–15 years		7(24.1%)
15–20 years		2(6.9%)
20 + years		8(27.6%)
Type of Practice (select all that apply)		
Oncology		17(58.1%)
Palliative Care		10(34.5%)
Hematology		6(20.7%)
Stem Cell Transplant		3(10.3%)
Cellular Therapy		3(10.3%)
Clinic Setting		
Inpatient		4(13.8%)
Outpatient		18(62.1%)
Both		6(20.7%)

*BiTE* bispecific t-cell engager, *BiKE* bispecific killer cell engager, *TriKE* trispecific killer cell engager, *MD* Medical Doctor, *DO* Doctor of Osteopathy, *APRN* Advanced Practice Registered Nurse, *PA* Physician Assistant

The majority of patients were 51–65 years of age (*n* = 14, 48.3%), female (*n* = 25, 86.2%), non-Hispanic (*n* = 19, 65.5%), white/Caucasian (*n* = 19, 65.5%), with bachelor’s degree reported as their highest level of education (*n* = 13, 44.8%). Patient participants had received a variety of cancer care treatments including chemo (*n* = 16, 55%), surgery (*n* = 15, 52%), radiation (*n* = 14, 48%), immunotherapy (*n* = 7, 24%), palliative care (*n* = 7, 24%), stem cell/bone marrow transplant (*n* = 1, 3%), and other treatments (*n* = 6, 21%) including targeted therapy, Arimidex, lymphedema treatments, aromatase inhibitor, and clinical trials. Clinician participants were mostly between 36–50 years of age (*n* = 17, 58.6%), female (*n* = 25, 86.2%), non-Hispanic (*n* = 27, 93.1%), and white/Caucasian (*n* = 27, 93.1%). Clinician participants included 9 (31%) physicians, 6 (20.6%) advanced practice providers, and 14 (48.3%) registered nurses. Clinicians primarily worked in outpatient settings (*n* = 18, 62.1%), in oncology (*n* = 17, 58.1%), palliative care (*n* = 10, 34.5%), or hematology (*n* = 6, 20.7%). Most clinician participants had 5–10 years in practice (*n* = 10, 34.5%), although several had more than 20 years (*n* = 8, 27.6%).

Table 3 reports responses about the overall opinion of the Information Blocking Rule.

**Table 3:** Overall opinion of the Information Blocking Rule

**Variable**	**Patient (*N* = 27)** Number(%)	**Clinician (*N* = 29)** Number(%)
Overall Opinion	SD = 2.553	SD = 1.227
Strongly support	13(44.8%)	2(6.9%)
Somewhat support	2 6.9%)	9(31%)
Neutral	1(3.4%)	4(13.8%)
Somewhat oppose	4(13.8%)	10(34.5%)
Strongly oppose	0(0.0%)	4(13.8%)
Not sure	0(0.0%)	0(0.0%)
Never heard of it	7(24.1%)	0(0.0%)

*SD* Standard deviation

Overall, more patient participants (*n* = 15, 51.7%) somewhat or strongly supported the policy than clinician participants (*n* = 11, 37.9%). Additionally, 14 clinicians (48.3%) were somewhat or strongly opposed to the policy while only 4 patients (13.8%) reported opposition. Seven patients (24.1%) indicated they had never heard of the policy, while no clinicians indicated they had not heard.

During interviews, all participants were asked what they want policy makers to know. Themes from these responses, from each group, are presented below.

### Patient themes

Patient responses to what they would like policy makers to know centered around 4 themes: (1) Information Blocking Rule is a good thing, thank you!; (2) everyone’s different; (3) patient and clinician should decide; (4) could have been implemented better. Each theme is expanded on below. Additional quotes for each theme can be found in Table 4.

1. ***Information Blocking Rule is a good thing, thank you!***

**Table 4:** Patient themes and illustrative quotes

Theme	Quotes
Information Blocking Rule is a good thing, thank you!	*[I would say] well done. Because it was time that we can taking charge of our health. It's time to get access to that one, no use being area that you cannot read it, so you don't know what's going on. (P33)* *I would say thank you, because I appreciate the right to have access my information. And what it makes me think of is the first time around [with my previous cancer] and the pathology slides and what happened to them. Then fast forward, 11 years, and I get this diagnosis, Eastpoint University is now trying to track down the slides. They want to see the actual slides and not just the report from 11 years ago. Oh, well, guess what? That facility disposed of them after 10 years. But nobody asked me, do you want us to get rid of them? But then I don't know how long do you keep pathology. That's a whole different thing, but that's what it makes me think of was, you didn't have the right to get rid of that without letting me know. (P7)* *[I would say] Thank you for being compassionate to cancer patients that would get a scan and wait for two weeks to get results, because it is extremely stressful. And if it forces doctors to make these appointments closer together and be more patient proactive, bless you. (P14)* *[I would say] good job because you're making it so us, as patients, can read what the doctors are saying to us clearly and accessibly and I like it. I like it. I've even had a doctor say, "Well you know you're going to read my notes, right?" And I'm like, "Yeah." I even read my oncology notes when I leave infusion where it's two sentences. Yes I access. She left without paperwork. She's the access. I read it just to make sure. (P11)* *So to have that available to you so you can… I mean, if you want to vet it yourself and you want to go online, there are some really great sources online to really give you more information about whatever you've been diagnosed with. You know how to spell it, you know what stage its in, you know all the extra little things that they put after it like if this has a T79EM after it, that's different. If it says EGFR and ALK inhibitors, I can't remember that from my appointment or that's something I could start researching….I mean, it probably frustrates the doctor if someone comes in and they get it from a source that's maybe not the most reputable source. I'm sure that's frustrating for the doctors. But there are also people that are highly educated that can go in there and they can discern information from good and bad and they can come up with some really great questions for their doctors as well because doctors treat a lot of diseases and a lot of different people and they might not be up on everything. (P13)* *I think, I think most people want to know and want to be involved and you know, some people just want the doctor to say show up at this time and you know, we're done, you know, we're gonna do what we're gonna do, but most people, and I'm always amazed when I talk to people who, um, maybe socioeconomically or educationally, I wouldn't expect them to be able to discuss it at a, that level, but they've, they may, they may not know a lot of things, but boy, they can talk to you about their disease in great detail and they know a lot of stuff. And I think it's a matter of, you know, you, you educate yourself on the things you need to know. (P31)*
Everyone’s different	*So I think to those lawmakers [I would say], it's a personal preference and that they should honor that for those people who can accept it or who cannot accept it. (P35)* *I'm going to tell them that in any situation, you're going to have differing opinions. And I would just… I think politicians in government at this point, I don't have a lot of confidence, and the fact that I feel like it's all about self-interest and not really about people and about human emotion and empathy. I would just always encourage that before decisions are made, there's consultation not just by clinicians or policymakers, it's about people at the end of the day. We are people. And I get why the disclosure is… Thought that was a great idea. I can see why someone would do that, and I could see several people that probably really love that, but that doesn't speak to everybody, and I think it's a choice. (P6)* *And so that's what I would ask policymakers. "Is this something that applies to your life? Do you get test results? Do you want your test results coming to you immediately without being informed or…" You know what I mean, "Do you want bad news coming to you instantly?" So I look at it in that way, basically treat people the way you want to be treated. (P16)* *I think they get the information out in a timely fashion. I don't need if they need to be told to do that. Physicians are of the mindset that people need to know. I don't really think we needed a policy for you to tell doctors to tell us what we need to know. Because I felt like the information I'm getting, I get it in a timely fashion. So I don't think it's really necessary, but I don't make the policies but I think, and then in some instances, some people may feel they're not getting it, where I don't feel that way. So I would say to them, "I don't think y'all need to be told how to disseminate information to your patients." (P17)*
Patient and clinician should decide	*Maybe with the clinicians and the patient privacy, it has to be a personal … Maybe that physician says to that patient, "I know this is going to cause you a lot of anxiety. We're going to make an appointment. You're going to come in and we're going to have that face-to-face because you always sometimes think it's the worst." And especially, if you don't understand, our minds just naturally go, "Oh my God, I'm dying," or something. (P35)* *I think the providers should share with us as much information as they have, and as they know that we can understand and absorb and respond to, to do whatever we need to do to make it better. (P22)* *I like it but I… My blood work… Some simple things like blood work to me is okay. But I think if the doctor need to call you in and talk to you one on one, you don't need to have it from MyChart… It might say that the time I might have six months to live. I don’t think I would want to hear something like that on MyChart. (P15)* *Before any negative information is put out, maybe the patient and the provider get together before and discuss it before it's provided. (P34)*
Could have been implemented better	*Transparency is great. I'm all for it. But it's the logistics, it's the details. It's the people who are the ones who are really getting the impact and the ones who really should be deciding like, "Hey, do we really need to be pushing all this out instantaneous like this?" Is it doing more harm or is it doing more good? To be decided. (P10)* *What was the intent behind making that rule?* *Interviewer:* *Yeah. That's a good question. Are you asking me or is that what you would ask them?* *Participant:* *Both maybe. If you know the answer I'm asking you, but if you don't then yeah, I'd ask them that. What was the intent behind it? (P12)* *I don't know what things were like prior to the law because I was a healthy person. I wasn't needing to look at things frequently. I could easily wait a week for my doctor to call me back or if I had a physical or something. So I think probably like a lot of laws or whatever, it just needs some tweaking and improvement. Don't want to go backwards, but it's probably been out there long enough that we all know what probably needs to be addressed. (P19)* *Things like access to that and records, I love seeing the progress. I think the legislators are making progress and I've been there enough times to know. I had images of my head of how that whole process was going to work and they were all wrong. What I found was some incredibly smart, passionate people trying to maneuver in this really screwed up system. They're really trying to help make healthcare better. I believe the legislators, as a whole, are really trying but I think we have things that are just institutionally in place that are just completely screwed up and I don't know that it'll ever really get fully solved but what I want is for everyone to have access to the finest care without barriers. (P4)*

Several patient participants in our study wanted policy makers to know that the Information Blocking Rule was a good thing in their minds. Several patients stated that they would want express gratitude to policy makers for creating the Information Blocking Rule, because it is their health information, and they have a right to it. As one patient shared:

*“I think it’s a good thing. I’d say thank you for doing it because it shouldn’t be a secret. I mean, people are people, and they’re [clinicians] not gods.” (P13)*

Many patient participants expressed gratitude for the Information Blocking Rule, because it empowered them by speeding up when they received information and made them feel a part of the team.

*“I would say thank you, because I really like to be able to utilize that whole area of things. Just being able to see results. I mean, I know what numbers on my labs to worry about and not worry about, and it’s a comfort […] I just like getting the news as quick as I can…It makes me feel empowered […] a part of the team of what is going on with me.” (P9)*

Patients also shared how having access to clinical notes and test results provides them with the opportunity to educate themselves about their cancer and treatment.

*[I would say] “Thank you. Really I- I’m grateful for it. […]I just think anybody who is struggling with a diagnosis can, can slowly learn how to be more involved in their care and how to make those decisions. […] And I think even though that learning curve may be a bit of a shock to people in the beginning, I think it’s very empowering in the long run so I’m grateful for it.” (P31)*

(2) ***Everyone’s different***

While many patient participants had a positive view of the Information Blocking Rule, they also acknowledged the fact that everyone is different in terms of the types of information they want to receive and how they want to receive that information. This is largely centered on the fact that with cancer, patients’ worlds revolve around receiving emotionally charged information, and as one patient explained,

“*it’s good if the information you’re being provided is positive. But if it’s some kind of negative information, I don’t feel it’s good there.” (P34)*

As such, many patients expressed that it should be a personal decision rather than one made through legislative policy. As one patient explained:

*“Personally, I think it’s a good thing, but for a person that does have maybe a lot of anxiety, it may not be a good thing. I think it’s a preference person to person. If that person is willing to want that information, they should be having to access. If it’s a person that maybe is having a lot of issues and it’s not healthy, mental or physically for them, that should be their choice. I think it should be the people’s choice. The lawmakers are not the ones walking in those shoes. It’s the patients that are walking in those shoes.” (P35)*

A couple of patients had the traumatic experience of learning they had a new cancer through a patient portal notification, something they wanted policy makers to think about. As one of the patients said:

*[I would ask policy makers] “have they ever received bad news and they can’t talk to their doctor about it? If they ever had a loved one who has received bad news online and they had no one to talk to about it.” (P3)*

Another patient shared how Open Notes were good for them in navigating their cancer care, but that it was not a good experience for their friend:

*“I had a friend who recently got a cancer diagnosis, and I would say she’s a […] worrier […] She called me one night and said, “Can I come over?” It was late and I was like, “Yeah, sure.” And so she said, “Well, I just got my lab results and it says this, this and this.” Her husband was like, “What do we do?” […] She saw those results and she was stressing out […]So in that case,[…]for her, it [receiving test results through the portal] wasn’t a good choice.” (P35)*

(3) ***Patient and clinician should decide***

Because “everyone is different” and a close relationship that is often formed between patients and clinicians in cancer care, several patient participants expressed that the decision about what information is shared through the patient portal and when should be decided between the patient and the clinician, rather than by a policy. As one patient explained:

*“I would say that they need to allow my healthcare team to decide what is best to send to me and for me to decide what is best to be sent to me, not them. […] The people who are being affected by it need to be the ones to decide, not the head honchos who think they know everything.” (P10)*

It was evident that many patients had developed trusting relationships with their clinicians, that their clinicians knew them and their situation best. They wanted to make informed decisions in consultation with their clinician, and some felt that the Information Blocking Rule took away that ability.

*“Don’t take the professionalism away from the doctors. […] I mean, she’s had to break news to me like that before. And it broke my heart, but when it broke my heart, I could see that it bothered her too. And that’s, and I hate saying, but that’s the way it’s supposed to be. I mean, we’re not dogs, […] you know what I’m saying?” (P32)*

(4) ***Could have been implemented better***

While all patients were able to identify what they would want to say to policy makers about the Information Blocking Rule after the interviewer explained what it was, only one patient had even heard of the Information Blocking Rule prior to the interview, indicating a clear lack of dissemination planning or planning for policy implementation. As one patient shared:

*“I personally was not aware of it until suddenly it [clinical notes] just came out and then same thing with test results just started coming out. When I was sitting in my patient room, waiting for my doctor to come in, I have labs first and then I go to my room and I sit there and I wait and suddenly, “Hey you, a test result.” It’s like, what? And I’m like, “Oh my labs are here, but I haven’t seen my doctor. What am I supposed to do with this?” (P10)*

Another explained how the policy sounded good in theory, but had concerns over how it was being implemented:

*“I feel like I would tell them how much of a can of worms they opened. But in a good way, I think it’s a good […]I get the perks of it and I get the benefits of it, but I wish that it would’ve been maybe rolled out more carefully or more slowly to allow health providers to kind of catch up and know the nuances and know how to navigate kind of the unknown terrain because I feel like ultimately that could be harmful to patients.”(P5)*

Finally, one patient expressed the importance of making sure the Information Blocking Rule did not cause increased burden on healthcare clinicians, particularly in the setting of cancer.

*“Make sure that the providers are not overloaded, because that’s one of things these doctors are getting burnt out. They just have so many patients they can’t keep up […] you go in there 15 minutes, then you go onto the next. Your provider needs to have the time to sit down and discuss these with you and don’t overwhelm these doctors, particularly with something as important as cancer treatment. (P30)*

### Clinician themes

Clinicians were also asked what one thing they would say to the policy makers responsible for the Information Blocking Rule. One respondent did not know what they would say, but everyone else had clear messages to send policy makers. While some of our participants acknowledged benefits of the policy for patients, most responses centered around unintended negative consequences of the policy—for both patients and clinician—and centered on the following themes: (1) cancer is different; (2) instant release is causing harm; (3) add safeguards, and (4) increased stress and workload. Illustrative quotes are included in the text and additional quotes for each theme can be found in Table 5.

1. ***Cancer is different***

**Table 5:** Clinician themes and illustrative quotes

Theme	Quotes
Cancer is different	*Much of what we deal with is sensitive information. Cancer is a very serious thing. It’s not just I’m going in for my cardiac score or whatever.* (C2) *When I get labs drawn or get a note from my primary care physician, […] that is a totally different report than telling somebody, *via* a path report with no communication that, “Oh, by the way, your cancer’s back.” or, “Oh, by the way, your MRI of your brain shows that you have brain metastases.” Like it’s just the level of recording is just so so different. […] That’s what I would tell people is that you can’t take a primary care physician’s or provider’s results and assume that it’s going to be the same level of comfort or anxiety for somebody that is already anxious about what their cancer is going to do.* (C5) *It’s a good idea in theory, but it’s not been applied practically. With so much of medicine, it needs a more deft hand than they’ve given it. And perhaps sharing with, listening to some frontline [oncology] clinicians, whether that be, inpatient clinicians or outpatient clinicians, specialties, or maybe some frontline clinicians and getting their take on the nuances of it would help. Rather than just a one size fits all [types of healthcare] type of policy.* (C23) *I wish you would’ve checked with providers before it was instituted because I don’t think it’s appropriate in the oncology world that pathology results, that patients find out they have cancer from their electronically result before they talk to a provider. It may be appropriate in internal medicine or family practice type setting or endocrinology type setting, but I think it adds to the anxiety in a, and I can only speak from this, but maybe more of the sub-specialties in situations.* (C18)
Instant release causing harm	*I would say to them, for cancer patients, if you had a cancer and you got a test result that you weren’t expecting and you’re able to see your result, how would you feel if you read in a report that you have cancer and nobody’s prepped you or talk to you about it? What is the impact on your life? Because I feel like that would be shattering. I mean, it really would. It would be devastating to read in a report, oh, here you have cancer everywhere. You have cancer in your lungs, in your bones, in your brain, in your abdomen and you thought you had like a benign cyst, but no. So now you’re sitting and wondering and panicking because Dr. Google says all of these things, but nobody’s talked to you about what this result means. Nobody’s been able to go over it with you before you’ve read it and come to all these conclusions because where does your mind go automatically? This is the death sentence.* (C29) *Well, I guess I understand where a patient owns their chart. Right? It’s their information. They should have access to their information. But some of the information should be seen by a doctor first, before releasing to the patient so that there are no misunderstandings. That’s what I think there’s no fear. I mean, there’s always going to be fear when you get a scan, but there’s no misinterpretation of results. […] That’s the biggest issue. And I think that’s, I don’t know. It’s kind of mean. It is. It’s kind of mean to do to them*. (C12) *So I think the sentiment is good and I appreciate it, but I do think there is just so much sensitive, complicated medical information that unleashing that to people who are not capable of really understanding it could be quite dangerous and harmful.* (C26) *I would say that there’s a reason we have physicians that go to school for 12 years and are able to read and figure out scans and labs and be able to embrace that for patient, if there’s bad news or. I think there’s a reason we see physicians is because they’re able to interpret that things. And I don’t think patients should get that [test results] first [before the physician]. It’s dangerous, especially if you were a new diagnosis*. (C33) *I would say that the thought and intention behind it is great. I would take a step back and put yourself in a patient’s shoes, even being a nurse and getting my regular yearly labs done myself, it’s already anxiety-provoking and I know what the labs mean. And so, putting in yourself in a patient’s shoes that might not have any educational background at all, and then seeing lab results, biopsy results, everything like that, I think it can be anxiety-provoking, hard and sometimes can lead patients to have depression and sit on those results for some time before they’re able to talk to a provider.* (C19)
Add Safeguards	*My suggestion would just be certain things like imaging or surgical pathology would have more of a window or grace period for the physician to have an opportunity, to go over it before it was released*. (C34) *I think that there is a lot of unnecessary harm that we are causing patients right now. With probably some tweaks to the system we could improve on. I think a delayed release of the labs, pathology scans, which would probably have to be across the board.* (C2)
Increased stress and workload	*I mean, I think initially it added more anxiety to all the providers, because I think people… I mean, as a provider, we’re nervous about what we’re going to get. Are we going to get barraged with phone calls all day long?* (C11) *And then I think too, it is a very real thing that this additional stress has put a lot of even more burden and expectation on providers as a whole. And I know that we’re all, in this country, stressed out, but I am amazed at the number of colleagues who were maybe around retirement age, who are just like, “I’m out.”* […] *I definitely have a higher number of patient messages now. And it does add to my workload. And so it’s contributing more to physician burnout, provider burnout, I probably should just say, it’s everybody. I know that my nurse, for example, who was an amazing person, she even shortly after the CURES Act said, “For the first time ever, I am not enjoying my job as much now.”* (C2) *And I think if I were on the patient side, I think it could be very helpful. I think though, that there are also definitely drawbacks and the freedom to look at your information at any given time is coming with some downsides, probably some, a lot of anxiety for patients and families, a lot of extra work for physicians who are trying to appease patients, explain things*. (C26) *I feel like we should be transparent in getting our information to our patients and families. I don’t really have any negatives. I think it’s just working around how to be better about how to approach this now that it’s here*. (C15) *But most physicians don’t ever want to change anything, even if it’s to their benefit, because they’re so used to whatever it is that they’re doing, that they’re rutted in that system and changing it in some ways it’s going to involve at least a short-term period of pain before things get better*. (C9)

Participants in our study wanted policy makers to consider how the comprehensive policy was implemented across very different healthcare specialties. Participants were clear that they felt cancer care was different, and that this difference should be recognized.

*“I think sometimes policies or acts like this that could potentially affect all people don’t necessarily apply to the cancer world. And it’s just a whole another world that we’re living in and that patients are living in.”* (C17)

Respecting the ways that cancer is different, participant clinicians also wanted policy makers to tailor the policy according to these unique aspects of oncology.

*“You can’t put all of healthcare into a single pot. A piece of primary care notes and primary care labs are going to be very different for patients than oncology patients, mental health patients. You can’t lump all of healthcare together. So it needs to be looked at differently.”* (C30)

Respondents wanted policy makers to know that not all healthcare specialties are the same, and that they should not be treated with a “one size fits all” policy. In particular, they felt that the sensitive nature of a lot of oncology information should be handled with care, and with input from clinicians in the respective specialties. This leads into the next theme about unintended consequences of the instant release of test results.

(2) ***Instant release is causing harm***

Numerous clinician participants felt very strongly that policy makers should know that instant release of information in oncology is directly causing harm.

*“I think that if we’re here to take care of the patients, I feel like this is not very good patient care. We’re essentially handing them on some patients, a death sentence and the computer is telling them that instead of somebody that really cares.”* (C27)

Clinicians were very concerned about the anxiety and distress finding out sensitive results could cause for patients with cancer.

(3) ***Add “safeguards”***

Due to concerns about patient harm, participants advocated for policy makers to consider adding “safeguards” around the release of information in order to mitigate this harm:

*“I think I would share just my general sentiment that I think that the intention is good of being able to share more information with patients, but that there are a lot of safeguards that need to be put in place that are not there currently. So in terms of potentially delaying critical results or delaying a new catastrophic diagnosis or instances where we need to be able to communicate in the electronic record with each other about important social issues or things like that, that may not be very palatable to patients to read that […] there are a number of safeguards that feel like they’re lacking*.” (C7)

As this last quote makes clear, while many cited test results as a key area needing safeguards, some (often palliative care clinicians) also identified notes with sensitive information as another circumstance needing safeguards. Some safeguards suggested in these instances were not including prognosis, being mindful of how to document difficult family conversations, and helping clinicians learn to be aware of medical terminology that could be misinterpreted or inflammatory (e.g., non-compliant, obese, substance abuse).

(4) ***Increased stress and workload***

Some participants did call attention to unintended negative consequences for their own stress and workload. One participant explained how the instant release of test results created stress by being caught off guard when speaking with patients who read them before they even had a chance to:

*“I don’t think there’s anything kind of more uncomfortable than reading along with the patient while you’re on the phone with them as they’re hysterical and you’re trying to figure out what the plan is.”* (C6)

Another shared the impact on their workload due to increased patient calls and the need for support:

*“if you’re going to ask a group of providers to be doing more, putting more work on them, then along with that should go the support to get it done […] start paying an oncologist 24/7 call to sit there and answer the questions of the patients who are freaking out, because of the rule they made.”* (C8)

Of note, several clinician participants acknowledged that while they originally feared that the policy would result in increased stress and workload, their opinion of it and its impact on them had improved over time.

*“I mean honestly, I feel like there was some anxiety before it happened, but I find it beneficial and I’m glad it exists.”* (C22)

One even recognized that it may be similar to previous policies, in which there is pushback at first but then it gets “ironed out.”

*“When HIPAA first came out—and it did iron itself out—but we had the same kind of trepidation at first. We’re like, this is never going to work. And it was a complete show at first, it didn’t work out well, but I think it took about five years and then it ironed itself out okay*.” (C23)

## Discussion

Our mixed methods study reported patient and clinician reactions to the Information Blocking Rule in cancer care and what they would like policy makers to consider. As our findings show, respondents generally had strong feelings about the Information Blocking Rule and important messages to relay to policy makers. As with other policies implemented into dynamic healthcare environments [16, 17], this change was met with a mixed reception.

Similar to previous research [18], patients in our study indicated stronger support of the Information Blocking Rule when compared to clinicians. Our findings add to a growing body of literature indicating overall patient support for shared clinical notes and test results [19, 20] with some concerns about increased confusion, anxiety, and regret [21, 22]. These survey findings were further confirmed and expanded on during qualitative interviews.

Patients emphasized gratitude for the Information Blocking Rule but also expressed how little they knew about it prior to its enactment, indicating a need for dissemination strategies to better inform patients. Conversely, all clinicians in our study indicated they had heard about the Information Blocking Rule when asked about it on the survey. However, several shared during interviews that they could not recall how they learned about it and did not fully understand its implications. Future studies that examine clinician education on the policy as one component of an implementation strategy to improve clinician understanding, support, and use are warranted.

Some clinicians in our study expressed that the Information Blocking Rule had increased their workload, stress, and potential burnout, a concern that was raised by at least one patient. This finding supports previous research indicating that clinicians worry about the potential for increased workload as a result of sharing their clinical notes and test results with patients [23–25]. Clinicians also explicitly iterated that cancer is different, and that the differences should be reflected in the policy regulations.

Several key areas of consensus emerged among patients and clinicians about the Information Blocking Rule in the cancer care setting. First, both patients and clinicians discussed the unintended stress and harm caused to patients from finding out sensitive health information (like a cancer diagnosis) through a portal and not from a trusted member of their health care team. This finding illustrates the importance of including patients who have serious illness or diagnoses such as cancer and their clinicians when developing and enacting policies that have the potential to have a significant impact on their well-being.

Second, both patient and clinicians agreed that policy makers should know that the Information Blocking Rule would benefit from more specification, or tailoring. Patients expressed the desire for tailoring in the form of individualization—emphasizing that each patient is different and has different preferences. Patient participants in our study wanted policy makers to consider that patients be able to discuss with their clinicians what type of information they wanted and how they wanted it delivered. Clinicians expressed a need for tailoring based on type of test result and potential information released—emphasizing that delaying critical results, such as imaging, pathology scans, and labs could prevent causing patients unintentional harm.

Our participants were clear that they believe the implementation of the Information Blocking Rule needs to improve in cancer care. However, the best way to optimize the policy is currently unknown. Updates to the Information Blocking Rule exceptions are ongoing. For example, withholding information due to concern that the information may cause patients anxiety was previously not an approved exception. The exceptions were recently updated to include patient harm [26] Thus, interpretation and implementation of the Information Blocking Rule likely varies across systems, signaling the need for continued education and dissemination.

Patients expressed a desire to make decisions about receiving sensitive information with their clinician. Other potential options could include discussing their information release profile with a specialized healthcare team member with expertise in patient portals and cancer care. Future research should assess the feasibility and satisfaction of potential implementation models. It is critical that we examine how best to tailor implementation of the Information Blocking Rule to retain its benefits without introducing unintended harm for patients with cancer.

To our knowledge, this study is the first to explicitly ask patients and clinicians in the cancer setting about their opinion of the Information Blocking Rule, and what they would like policy makers to know. Thus, our findings elucidate the on-the-ground realities of this policy in cancer care and provide suggestions for optimizing its implementation.

Our study also has limitations. First, while we included patients with a variety of cancer types and a variety of professionals working in a variety of cancer care areas, all participants were from one, large academic cancer center in the Midwestern U.S. Thus, findings may not be generalizable to patients and clinicians in other cancer care settings or regions. Additionally, participants in our study were mostly female and white/Caucasian. No participants indicated they were transgender or non-binary. None were from races other than white/Caucasian, Black/African American, and few who reported they were Hispanic. Thus, exploring potential differences by gender, ethnicity, and race were not possible.

## Conclusions

In April 2021, the 21^st^ Century Cures Act Interoperability and Information Blocking Rule went into effect, requiring health care clinicians to give patients rapid access to all clinical notes and test results. Our study investigated the responses to this policy from patients and clinicians in cancer care. To optimize the implementation of the Information Blocking Rule and minimize unintended harm, future research should test implementation models and continue to include on-the-ground data from the individuals directly impacted by the policy.

## Supplementary Information

**Additional file 1.** Supplemental Surveys and Interview Guides.

## Data Availability

The datasets generated and/or analyzed during the current study are not publicly.available due to privacy concerns and in accordance with data protection measures from our institutional review board-approved protocol but are available from the corresponding author on reasonable request by email: hnelson-brantley@kumc.edu.
