# Supplementary material for: Understanding the Cures Act Information Blocking Rule in cancer care: a mixed methods exploration of patient and clinician perspectives and recommendations for policy makers
Source: BMC Health Serv Res. 2023 Mar 6;23:216. doi: 10.1186/s12913-023-09230-z (PMC9990332; doi:10.1186/s12913-023-09230-z)
Supplement: Supplementary file 1 — Additional file 1. Supplemental Surveys and Interview Guides. [file 12913_2023_9230_MOESM1_ESM.pdf]

Patient Demographics

Please complete the survey questionnaire at your convenience. It is anticipated to take approximately 10 minutes to complete. This is a voluntary survey - you will not be identified through your responses to this survey. There may be questions that you do not feel comfortable answering and it is okay if you choose not to - there will be no consequences for not completing parts or all of the survey.

Thank you for participating in this important study.

Please select if you are a patient or caregiver.	◯ Patient<br>◯ Caregiver
Please select your age range.	◯ 20-35 years<br>◯ 36-50 years<br>◯ 51-65 years<br>◯ 66+ years<br>◯ Prefer not to say
Please select your gender identity.	◯ Female<br>◯ Male<br>◯ Transgender<br>◯ Non-binary<br>◯ Prefer not to say
Please select your ethnicity.	◯ Non-Hispanic<br>◯ Hispanic: Mexican, Mexican American, Chicano<br>◯ Hispanic: Puerto Rican<br>◯ Hispanic: El Salvadorian<br>◯ Hispanic: Other<br>◯ Prefer not to say
Please state how you identify racially (please select all that apply):	☐ White/Caucasian<br>☐ Black/African American<br>☐ Asian<br>☐ Native Hawaiian or Pacific Islander<br>☐ Native American/Alaskan Native<br>☐ Other<br>☐ Prefer not to say
Please state the other race(s) you identify as.	________________________________
Please state the highest degree of education you have completed.	◯ High School Diploma or GED<br>◯ Vocational/Technical Training<br>◯ Associate's Degree<br>◯ Bachelor's Degree<br>◯ Master's Degree<br>◯ Doctorate<br>◯ I did not complete high school or receive a GED<br>◯ Prefer not to say

Please provide the zip code where you live.

____

Please select the type of cancer care you are currently receiving, have received or plan to receive within the next 3 months (please select all that apply):

☐ Chemotherapy
☐ Radiation
☐ Surgery
☐ Palliative care
☐ Monitoring on a schedule
☐ Immunotherapy
☐ Stem cell/Bone marrow transplant
☐ BiTE/BiKE/TriKE treatments
☐ Tumor-infiltrating lymphocyte (TIL) treatment
☐ Other

Please list the other type(s) of cancer care you are receiving.

____

Please click submit below to move onto the next page of the survey.

Patient Opinions

1) What is your overall opinion of the 21st Century Cures Act Interoperability and Information Blocking Rule that requires health care providers (such as doctors and nurses) to provide patients with on-demand access to their clinical notes?

- ◯ Strongly Support
- ◯ Somewhat Support
- ◯ Neutral
- ◯ Somewhat Oppose
- ◯ Strongly Oppose
- ◯ Not Sure
- ◯ Never Heard Of It

2) How often do you look at or read the clinical notes that were written by your provider in MyChart (the patient portal)?

- ◯ Daily
- ◯ Weekly
- ◯ Monthly
- ◯ Never

The next set of questions require a response based on a scale of 1-100, with 1 being the least and 100 being the most. Please use the slider button to respond to the question.

3) How confident are you in your ability to understand notes that are written by your provider and shared through MyChart (the patient portal)?

1 50 100

(Place a mark on the scale above)

4) How much do you desire to see and use notes that are written by your provider in MyChart (the patient portal)?

1 50 100

(Place a mark on the scale above)

5) How comfortable are you with seeing and using notes that are written by your provider in MyChart (the patient portal)?

1 50 100

(Place a mark on the scale above)

The next set of questions require a response based on a scale of 0-100, with 0 being "not concerned at all" and 100 being "the most concerned" regarding each statement. Please use the slider button to respond to the question.

6) Not knowing how to access the notes.

0 50 100

(Place a mark on the scale above)

7) Not understanding the medical terms used.

0 50 100

(Place a mark on the scale above)

8) Privacy - such as others being able to get access to the notes without your permission.

0 50 100

(Place a mark on the scale above)

9) Having questions and not knowing how to respond (such as "Should I call the clinic?", "Should I wait for my next appointment?", or "Should I ask my family/friends?", etc.).

0 50 100

(Place a mark on the scale above)

10) Provider using language that may offend me.

0 50 100

(Place a mark on the scale above)

11) Receiving bad news.

0 50 100

(Place a mark on the scale above)

12) Receiving results that are difficult to understand after office hours and having questions.

1 50 100

(Place a mark on the scale above)

Patient Suggestions

From the list below, please select your HIGHEST concern you have.	◯ Not knowing how to access the notes<br>◯ Not understanding the medical terms used<br>◯ Privacy - others being able to get access to the notes without my permission<br>◯ Having questions and not knowing how to respond (i.e., should I call the clinic, wait for my next appointment, ask my family/friend, etc.)<br>◯ Provider using language that may offend me<br>◯ Receiving bad news<br>◯ Receiving results that are difficult to understand after office hours and having questions.<br>◯ I have no concerns
From the list below, please select your SECOND HIGHEST concern.	◯ Not knowing how to access the notes<br>◯ Not understanding the medical terms used<br>◯ Privacy - others being able to get access to the notes without my permission<br>◯ Having questions and not knowing how to respond (i.e., should I call the clinic, wait for my next appointment, ask my family/friend, etc.)<br>◯ Provider using language that may offend me<br>◯ Receiving bad news<br>◯ Receiving results that are difficult to understand after office hours and having questions.<br>◯ I have no concerns
From the list below, please select your LEAST concern.	◯ Not knowing how to access the notes<br>◯ Not understanding the medical terms used<br>◯ Privacy - others being able to get access to the notes without my permission<br>◯ Having questions and not knowing how to respond (i.e., should I call the clinic, wait for my next appointment, ask my family/friend, etc.)<br>◯ Provider using language that may offend me<br>◯ Receiving bad news<br>◯ Receiving results that are difficult to understand after office hours and having questions.<br>◯ I have no concerns
Do you have any other concerns regarding open notes that are not listed above?	◯ Yes<br>◯ No
Please state other concerns you have.	__________________________________
Please select which types of documents, notes or results you do not feel comfortable reviewing or do not fully understand what they say.	☐ Routine laboratory results (such as blood tests)<br>☐ Pathology laboratory results (such as tumor markers)<br>☐ X-rays or scans<br>☐ Pathology results from biopsies or surgeries<br>☐ Other<br>☐ I'm comfortable reviewing any notes in MyChart.
What other types of notes do you not feel comfortable reviewing or understanding?	____________________________

What would make you more comfortable seeing and using notes that are written by your provider in MyChart (the patient portal)? Please select all that apply.	☐ Instructions in MyChart on how to view the notes.<br>☐ Instructions sent by email about how to view the notes.<br>☐ If the note included information on how to respond if I have questions.<br>☐ Short video in MyChart explaining how to view the notes, and how to respond if I have questions.<br>☐ Provider explaining to me during an office visit what to expect when I read their note in MyChart and how to respond.<br>☐ A template that helps me understand medical abbreviations and common terms.<br>☐ Ability to opt out of receiving certain types of information (such as diagnosis, test results, life expectancy) in MyChart.<br>☐ Ability to provide suggestions for improvement.<br>☐ Class on how to view and use the notes in my cancer care.<br>☐ Other
What other suggestions do you have to make you more comfortable viewing and using notes?	__________________________________

Please click submit to move to the final page of the survey.

Patient FCCHL

Please complete the survey questions below. This porton of the survey is anticipated to take approximately 10 minutes to complete. There may be questions that you do not feel comfortable answering and it is okay if you choose not to - there will be no consequences for not completing parts or all of the survey.

Please think about the notes you have viewed in MyChart (the patient portal) to complete the questionnaire below.

When you read information in notes that your provider writes in MyChart (the patient portal) about your cancer care such as test results, diagnoses, medications, or instructions:

		Never	Sometimes	Often	Always
1)	Is the print too small to read, even with glasses or contacts?	◯	◯	◯	◯
2)	Are there words that you do not know or have never seen before?	◯	◯	◯	◯
3)	Is the information too hard to understand or you have to ask for help to understand?	◯	◯	◯	◯
4)	Does the information take a long time to understand?	◯	◯	◯	◯
5)	Do you need help from someone else to understand?	◯	◯	◯	◯

When you are finding or using information you obtained from notes that your provider writes in MyChart (the patient portal) about your diagnosis of cancer or cancer treatment:

		Never	Sometimes	Often	Always
6)	Do you seek additional information from different places (such as the internet, healthcare workers, family or trusted friends)?	◯	◯	◯	◯
7)	Can you find the information you wanted?	◯	◯	◯	◯
8)	Is the additional information you find understandable?	◯	◯	◯	◯
9)	Can you tell someone about your thoughts and concerns related to your health (such as a doctor, nurse, pharmacist, family or friends)?	◯	◯	◯	◯
10)	Can you use the information you are given or find in your daily life?	◯	◯	◯	◯

When you are given, finding, or using information about your cancer beyond what you received from your provider's notes in MyChart (the patient portal):

		Never	Sometimes	Often	Always
11)	Can you tell if the information applies to you and your diagnosis?	○	○	○	○
12)	Can you tell if the place you found the information is truthful or trustworthy?	○	○	○	○
13)	Can you tell if the information is true or correct?	○	○	○	○
14)	Can you use the information to make health related choices?	○	○	○	○

Clinician Demographics

Please complete the survey questionnaire at your convenience. It is anticipated to take approximately 10 minutes to complete. This is a voluntary survey - you will not be identified through your responses to this survey. There may be questions that you do not feel comfortable answering and it is okay if you choose not to - there will be no consequences for not completing parts or all of the survey.

Thank you for participating in this important study.

Please select your age range.	◯ 20-35 years<br>◯ 36-50 years<br>◯ 51-65 years<br>◯ 66+ years<br>◯ Prefer not to say
Please select your gender identity.	◯ Female<br>◯ Male<br>◯ Transgender<br>◯ Non-binary<br>◯ Prefer not to say
Please select your ethnicity.	◯ Non-Hispanic<br>◯ Hispanic: Mexican, Mexican American, Chicano<br>◯ Hispanic: Puerto Rican<br>◯ Hispanic: El Salvadorian<br>◯ Hispanic: Other<br>◯ Prefer not to say
Please state the origin.	__________________________________
Please state how you identify racially (please select all that apply).	☐ White/Caucasian<br>☐ Black/African American<br>☐ Asian<br>☐ Native Hawaiian or Pacific Islander<br>☐ Native American or Alaskan Native<br>☐ Other<br>☐ Prefer not to say
Please state the other race(s) you identify as.	__________________________________
Please select your professional licensure.	◯ Medical Doctorate (MD)<br>◯ Doctor of Osteopathy (DO)<br>◯ Doctorate of Nursing Program (DNP)<br>◯ Nurse Practitioner (NP, APRN)<br>◯ Physician Assistant (PA)<br>◯ Registered Nurse (RN)<br>◯ Licensed Practical Nurse (LPN)

Please select the number of years you have been in practice since completing your training.	◯ < 1 year<br>◯ 1-5 years<br>◯ 5-10 years<br>◯ 10-15 years<br>◯ 15-20 years<br>◯ 20+ years
Please select your area of practice. (Select all that apply.)	☐ Oncology<br>☐ Palliative Care<br>☐ Hematology<br>☐ Stem Cell Transplant<br>☐ Cellular Therapy
Please select your primary practice setting.	◯ Inpatient<br>◯ Outpatient<br>◯ Both
Please select the location of your primary practice.	☐ KU Hospital<br>☐ Westwood Campus<br>☐ KC Metro Community Clinic<br>☐ Topeka<br>☐ Great Bend
Please state where the clinic is located.	________________________________

Please click submit to move to the next page of the survey.

Clinician Opinions

13) What is your overall opinion of the 21st Century Cures Act Interoperability and Information Blocking rule that requires clinicians to provide patients with on-demand access to your clinical notes?

- ◯ Strongly support
- ◯ Somewhat support
- ◯ Neutral
- ◯ Somewhat oppose
- ◯ Strongly oppose
- ◯ Not sure
- ◯ Never heard of it

14) Do you currently share your clinical notes with patients through MyChart?

- ◯ Yes
- ◯ No
- ◯ Not sure

The next set of questions require a response based on a scale of 1-100, with 1 being the least and 100 being the most. Please use the slider button to respond to the question.

15) How confident are you in your patients' ability to understand clinical notes?

1 50 100

(Place a mark on the scale above)

16) How confident are you with sharing your notes with your patients?

1 50 100

(Place a mark on the scale above)

17) How confident are you in your patients' desire to see and use your clinical notes?

1 50 100

(Place a mark on the scale above)

The next set of questions require a response based on a scale of 0-100, with 0 indicating you are not concerned with the statement regarding open notes and 100 indicating you are the most concerned with the statement. Please use the slider button to respond to the question.

18) It is not the purpose of clinical notes.

0 50 100

(Place a mark on the scale above)

19) Patients will get confused.

0 50 100

(Place a mark on the scale above)

20) Patients will get upset, anxious, sad, and/or angry.

0 50 100

(Place a mark on the scale above)

21) Increased malpractice liability.

0 50 100

(Place a mark on the scale above)

22) Increased call volume/questions.

0 50 100

(Place a mark on the scale above)

23) Adapting your notes to be clear, succinct, and respectful of patients.

0 50 100

(Place a mark on the scale above)

24) Being able to communicate openly with other clinicians.

0 50 100

(Place a mark on the scale above)

25) Including necessary information for billing.

0 50 100

(Place a mark on the scale above)

26) Sensitive topics.

0 50 100

(Place a mark on the scale above)

27) More time documenting.

0 50 100

(Place a mark on the scale above)

28) I am worried about the timing and accessibility of test results (such as biopsies, scan results, or lab tests) being provided during out of office hours for my patients.

1 50 100

(Place a mark on the scale above)

Please click submit to move to the final page of the survey.

Clinician Suggestions

From the list below, please select your HIGHEST concern regarding use of open notes.	◯ It's not the purpose of clinical notes<br>◯ Patients will get confused<br>◯ Patients will get upset, anxious, sad, angry<br>◯ Increased malpractice liability<br>◯ Increased call volume / questions<br>◯ Adapting your notes to be clear, succinct, and respectful of patients<br>◯ Being able to communicate openly with other clinicians<br>◯ Including necessary information for billing<br>◯ Sensitive topics<br>◯ More time documenting<br>◯ I am worried about the timing and accessibility of test results (such as biopsies, scan results or lab tests) being provided during out of office hours for my patients.<br>◯ I have no concerns
Of the topics listed below, please select your SECOND HIGHEST concern regarding use of open notes.	◯ It's not the purpose of clinical notes<br>◯ Patients will get confused<br>◯ Patients will get upset, anxious, sad, angry<br>◯ Increased malpractice liability<br>◯ Increased call volume / questions<br>◯ Adapting your notes to be clear, succinct, and respectful of patients<br>◯ Being able to communicate openly with other clinicians<br>◯ Including necessary information for billing<br>◯ Sensitive topics<br>◯ More time documenting<br>◯ I am worried about the timing and accessibility of test results (such as biopsies, scan results or lab tests) being provided during out of office hours for my patients.<br>◯ I have no concerns
Of the topics listed below, please select your LEAST concern regarding use of open notes.	◯ It's not the purpose of clinical notes<br>◯ Patients will get confused<br>◯ Patients will get upset, anxious, sad, angry<br>◯ Increased malpractice liability<br>◯ Increased call volume / questions<br>◯ Adapting your notes to be clear, succinct, and respectful of patients<br>◯ Being able to communicate openly with other clinicians<br>◯ Including necessary information for billing<br>◯ Sensitive topics<br>◯ More time documenting<br>◯ I am worried about the timing and accessibility of test results (such as biopsies, scan results or lab tests) being provided during out of office hours for my patients.<br>◯ I have no concerns
Do you have any other concerns that are not listed in the questions above?	◯ Yes<br>◯ No

Please list any other concerns you have regarding open notes use.	______________________
What would make you more comfortable sharing your notes with patients?	☐ Additional training<br>☐ Standardized message for patients<br>☐ Customizable templates<br>☐ Prepare patients during their office visit for what they can expect to read about in the notes<br>☐ Ability to see how the note looks from my patient's perspective<br>☐ Ability to provide suggestions for improvement<br>☐ Added time delays for certain results or notes<br>☐ I already share notes and feel comfortable with doing so<br>☐ Other
What types of results or notes would you prefer had a timed delay? Please select all that apply.	☐ Pathology results from biopsies/surgeries<br>☐ Scan results<br>☐ Laboratory results such as tumor marker<br>☐ Other laboratory results<br>☐ Other
What other types of laboratory results would you prefer had timed delays?	______________________
What other types of notes and documents would you prefer had timed delays in delivery?	______________________
What other suggestions do you have to make you more comfortable?	______________________

Patient Interview Guide

1. Could you begin by telling me a little bit about your cancer care at KU, like when you started coming to KU, what type(s) of cancer care are you receiving?
2. Tell me about your experience with reading notes that your providers write about your cancer care visits through MyChart.
    a. Probes: Has anyone communicated with you about this? When were you first aware that you could access their notes? How did you find out? Can you share an example of a success you've had? Of a challenge you've experienced?
3. Have you ever looked at your blood counts [leukemia patients], protein levels [myeloma], scans [patients with solid tumors], or other test results in MyChart?

    <u>If yes, ask:</u>

    a. Can you recall how you felt when you looked at the results? Did you find them reassuring, frightening, confusing, etc.?
    b. What, if anything, did you do to try to get clarity (i.e., look up words on the internet, call a friend with medial training, call your provider)?
    c. In what way(s), if any, did your interpretation of the result(s) change when you heard the results from your healthcare team?

    <u>If no, ask:</u>

    a. Can you share how you think you might feel when looking at test results in MyChart? Would you find them reassuring, frightening, confusing, etc.?
    b. If you had questions or concerns about what you discovered, what might you do (i.e., look up words on the internet, call a friend with medial training, call your provider)?
4. How often do you read your provider's notes in MyChart? Test results?
    a. Probe: What are the reasons why you do/don't read the notes? Test results? Have there been any challenges? How have you worked around them?
5. Tell me about the benefits you've had [or can imagine if not reading them], if any, from reading provider notes in MyChart. From reading your test results.
    a. Probe: what things have you found helpful [or can imagine being helpful if not using/reading]?
6. What do you/could you do with the information you read?
    a. Probe: Do/would you look things up? Who, if anybody, do/would you share the note with? And why?
7. In what ways, if any, has reading your provider's notes and/or test results impacted you personally [or could you imagine it impacting you if not reading/using]?
    a. Probe: The way to think about your cancer diagnosis? Treatment? The way you engage in your care?
8. Have you ever contacted your provider after reading a clinical note or test result? If so, why? If not, why? How soon after? How satisfied were you with the response? If not currently reading notes or test results, how do you imagine you would respond if you read information that you were not certain about or were surprised to see?
9. What do you want your provider to know in regard to sharing information about your cancer treatment, diagnoses, etc. through MyChart?
    a. Probes for patients currently using: What is working well? What suggestions do you have for improving it?
10. Are there things or information you would rather not learn about for the first time through a provider note in MyChart? Through a test result?

   a. Probe: If you were to receive bad news, how would you feel? In what way, if any, would your reaction be different if you heard the news in person from your provider?

11. Are you familiar with the 21st Century Cures Act Interoperability and Information Blocking Rule? [if no, provide brief overview]. How do you feel about the policy?
    a. Probe: What, if anything, would you want your legislator to know? If you could share anything about this new policy, what would it be?
12. Is there anything else that you would like to share that we haven't discussed today?

Clinician Interview Guide

1. Could you begin by telling me a little bit about your role and how long you have worked in [oncology, palliative care, both]?
2. Tell me about your experience with clinical notes and test results being made available to patients through MyChart.
   a. Probes: Has anyone communicated with you about this? When were you first aware that your clinical notes were being made available to patients? How about test results? How did you find out? Can you share an example of a success you've had? Of a challenge you've experienced?
3. Do you have a sense of whether patients read your clinical notes and/or test results in MyChart and what they do with the information?
   a. Probes: Can you share a specific example?
4. In your opinion, what has been the impact on patients viewing your notes and/or test results in MyChart?
   a. Probes: What are they sharing with you? If you have observed positive outcomes, can you share a specific example? If you have observed negative outcomes, can you share an example?
5. What impact has it had on you and your interactions with patients?
   a. Probes: What changes, if any, have you noticed in the way you write your clinical notes? The way you view your patient? The length of time you spend writing notes? The speed in which you contact patients once results are known?
6. Are you familiar with the 21st Century Cures Act Interoperability and Information Blocking Rule? [if no, provide brief overview]. How do you feel about the policy?
   a. Probes: What do you want policy makers to know? If you could say anything to them about this new regulation, what would it be? In what ways, if at all, has your process of informing patients of diagnosis or discussing test results, treatment options and regimens changed now that the policy is in place?
7. What concerns you, if anything, about patients reading your notes in MyChart? About patients reading test results?
   a. Probes: How do you feel about the possibility of patients discovering errors in your notes? How often has this occurred in the past month? Past year? Since your organization started sharing clinical notes in MyChart?
8. In your opinion, what are the benefits of patients reading notes in MyChart? Patients reading scans or lab results?
   a. Probes: Can you share a specific example?
9. If you could change anything about clinical notes being made available in MyChart, what would it be?
   a. Probe: What, if anything, could your organization do to improve open notes?
10. Is there anything else that you would like to share that we haven't discussed today?
